# Supplementary material for: MFF-dependent mitochondrial fission regulates presynaptic release and axon branching by limiting axonal mitochondria size
Source: Nat Commun. 2018 Nov 27;9:5008. doi: 10.1038/s41467-018-07416-2 (PMC6258764; doi:10.1038/s41467-018-07416-2)
Supplement: Supplementary file 1 — Supplementary Information [file 41467_2018_7416_MOESM1_ESM.pdf]

Supplementary Information

MFF-dependent mitochondrial fission regulates presynaptic release and axon branching by limiting axonal mitochondria size

Lewis & Kwon et al.

Supplementary Figure 1

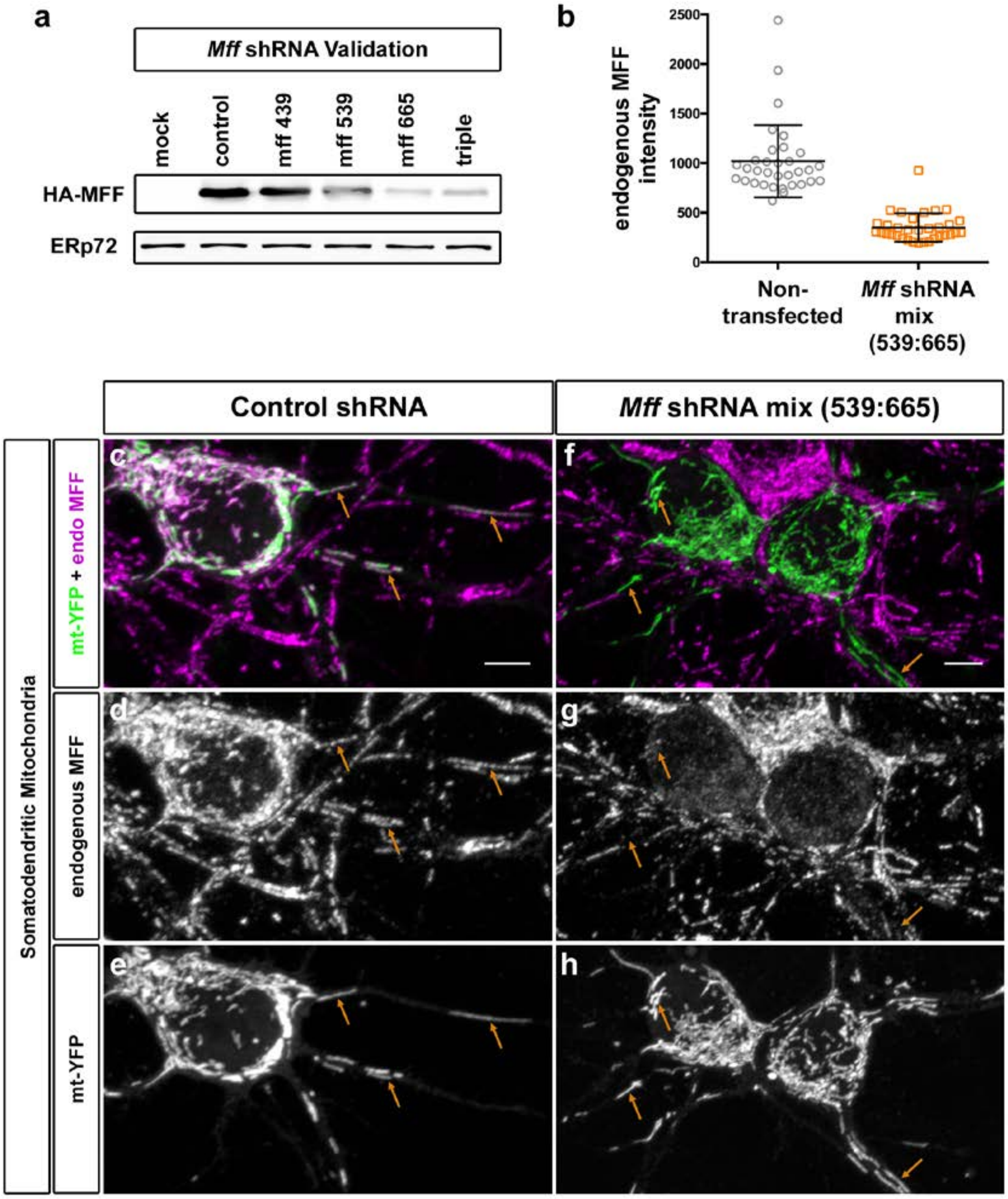

Supplementary Figure 1. Validation of *Mff* shRNA knockdown

(**a**) Western blot of individual shRNA knockdown efficiency via overexpression of HA-tagged mouse MFF (top) in HEK cells. Antibody against an endogenous ER protein (ERp72; bottom) was used as the loading control. (**b**) Analysis of knockdown efficiency of a 1:1 mixture of *Mff* shRNA's 539 and 665 by immunofluorescence of endogenous MFF levels. Mann-Whitney test: $p < 0.0001$. Data is from two independent immunocytochemistry staining from two independent cultures. (**c-e**) Representative images of a neuron electroporated with mt-YFP and control shRNA via EUE at E15.5, and stained at 7DIV with antibodies for GFP and *Mff*. (**f-h**) Representative images of neurons electroporated with mt-YFP and a (1:1) mixture of *Mff* shRNAs (539 and 665) via EUE at E15.5, and stained at 7DIV with antibodies for GFP and *Mff*. Orange arrows point to the same mitochondria in each set of images. Scale bars represent the following lengths: 5µm. Related to **Figure 1.**

Supplementary Figure 2

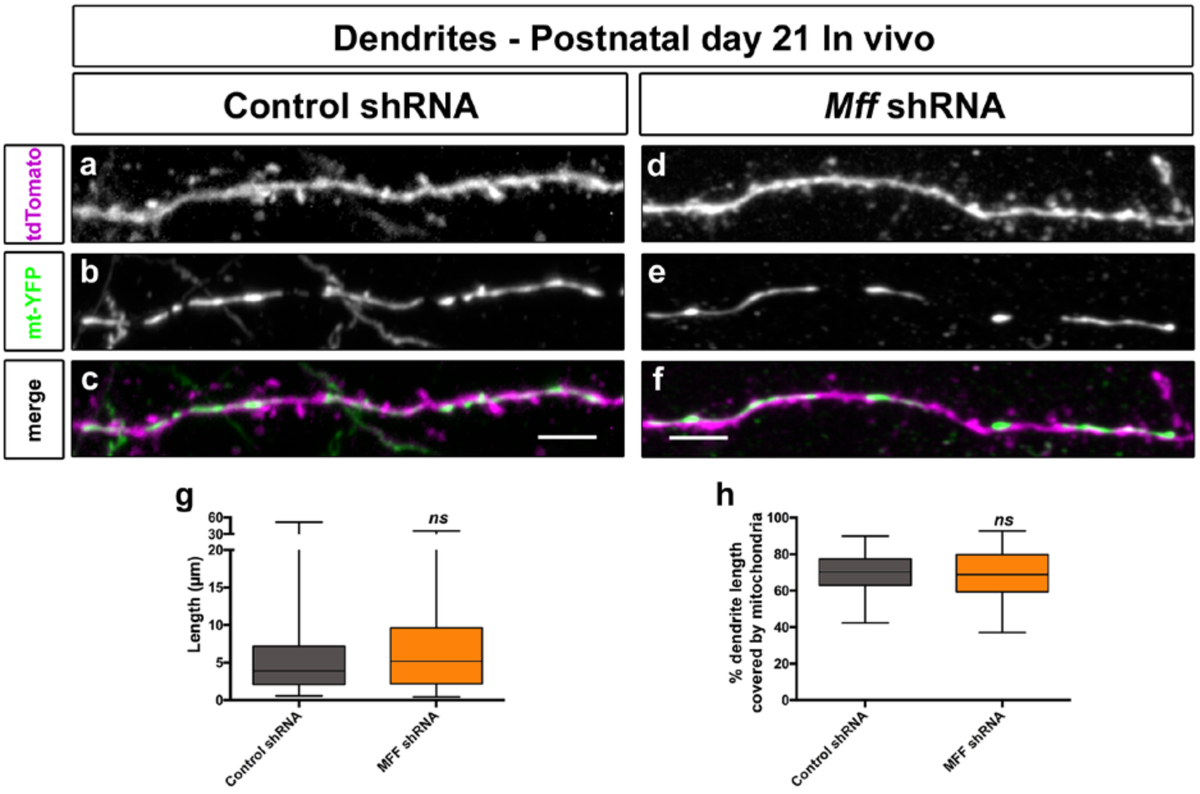

Supplementary Figure 2. Decreased MFF activity does not increase dendritic mitochondrial length

(**a-c**) Representative images of a dendrite from a neuron electroporated with mt-YFP, tdTomato and control shRNA via IUE at E15.5, and stained at P21 with antibodies for GFP and tdTomato. (**d-f**) Representative images of a dendrite from a neuron electroporated with mt-YFP, tdTomato and 1:1 mixture of *Mff* shRNA (539:665) via IUE at E15.5, and stained at P21 with antibodies for GFP and tdTomato. (**g**) Quantification of mitochondrial length in the dendrites. Data is represented at minimum to maximum box plots, with the box denoting 25th, 50th and 75th percentile. (**h**) Quantification of the percent of the dendrite occupied by mitochondria. Data is represented at minimum to maximum box plots, with the box denoting 25th, 50th and 75th percentile. $n_{control\ shRNA}$ = 26 dendrites, 267 mitochondria; $n_{MFF\ shRNA}$ = 19 dendrites, 146 mitochondria. n.s., p>0.05 according to Mann-Whitney test. Scale bars represent the following lengths: 5µm. Related to **Figure 2.**

Supplementary Figure 3

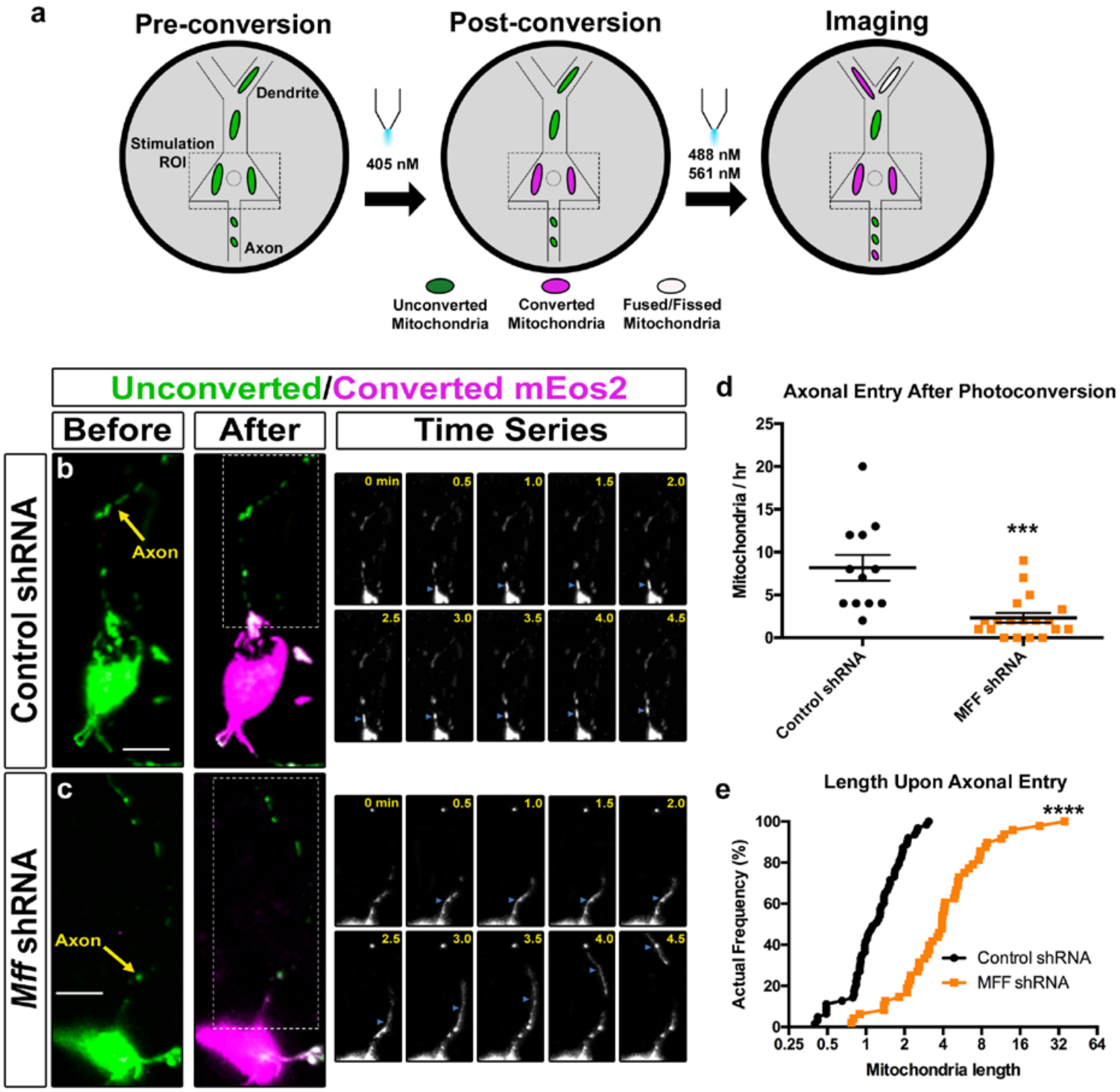

Supplementary Figure 3. Fewer mitochondria enter the axon but have increased length upon MFF knockdown
(**a**) Schematic of the imaging paradigm used for measuring axonal entry of mitochondria via mitochondrial-targeted mEos2. (**b**) Selected timeframes of mitochondria entering the axon of a 10DIV neuron EUE with control shRNA and mt-mEos2. (**c**) Selected timeframes of mitochondria entering the axon of a 10DIV neuron EUE with 1:1 mixture of *Mff* shRNA (539:665) and mt-mEos2. Fewer mitochondria enter the axon upon *Mff* knockdown, but are much longer. See supplementary movie 1. (**d**) Quantification of the number of axonal entry events per hour in 7-11DIV axons. Data is represented as a scatter plot with mean ± sem. ***p<0.001 for control vs. *Mff* shRNA. Mann-Whitney test. (**e**) Cumulative frequency of mitochondrial length upon axonal entry for control and *Mff* shRNA mediated knockdown in 7-11DIV axons.

**** $p<0.0001$ for control vs. *Mff* shRNA. Mann-Whitney test. $n_{control\ shRNA}$ = 12 axons, 63 mitochondria; $n_{MFF\ shRNA}$ = 19 axons, 48 mitochondria. Scale bars represent the following lengths: 10µm. Related to **Figure 3.**

Supplementary Figure 4

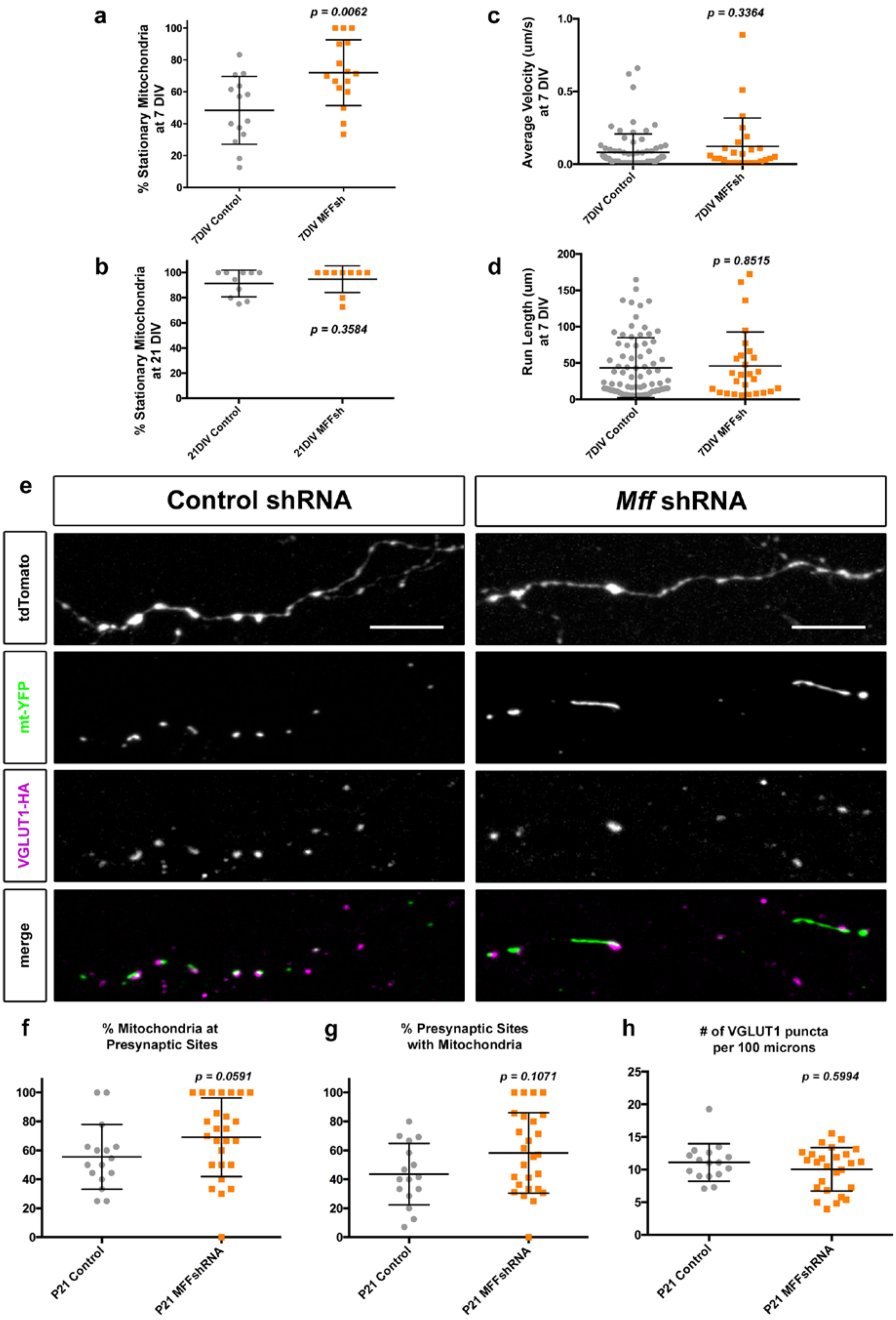

Supplementary Figure 4. Loss of MFF activity does not affect the final localization of axonal mitochondria at presynaptic sites

(**a**) Quantification of the percent of stationary mitochondria for 15min at 7DIV. Data is represented as a scatter plot with mean ± sd. $n_{control\ shRNA}$ = 14 axons; $n_{MFF\ shRNA}$ = 16 axons. See supplemental video 3. (**b**) Quantification of the percent of stationary mitochondria for 15min at 21DIV. Data is represented as a scatter plot with mean ± sd. $n_{control\ shRNA}$ = 10 axons; $n_{MFF\ shRNA}$ = 9 axons. (**c**) Quantification of average velocity for motile mitochondria in axons from (a) Data is represented as a scatter plot with mean ± sd. (**d**) Quantification of run length for motile mitochondria in axons from (a). Data is represented as a scatter plot with mean ± sd. (**e**) Representative images of an axon from a neuron electroporated with mt-YFP, VGLUT1-HA, tdTomato and control shRNA (left panels) or a 1:1 mixture of *Mff* shRNAs (539, 665; right panels) via IUE at E15.5, and stained at P21 with antibodies for GFP, HA and tdTomato. (**f**) Quantification of the percent of mitochondria at presynaptic sites. (**g**) Quantification of the percent of presynaptic sites with mitochondria. (**h**) Quantification of the number of VGLUT1-HA puncta per 100 microns of axon. Data is represented as scatter plots with mean ± sd. $n_{control\ shRNA}$ = 16 axons; $n_{MFF\ shRNA}$ = 26 axons. Mann-Whitney test; p values in figure. Scale bars represent the following lengths: 10µm. Related to **Figures 5 and 6.**

Supplementary Figure 5

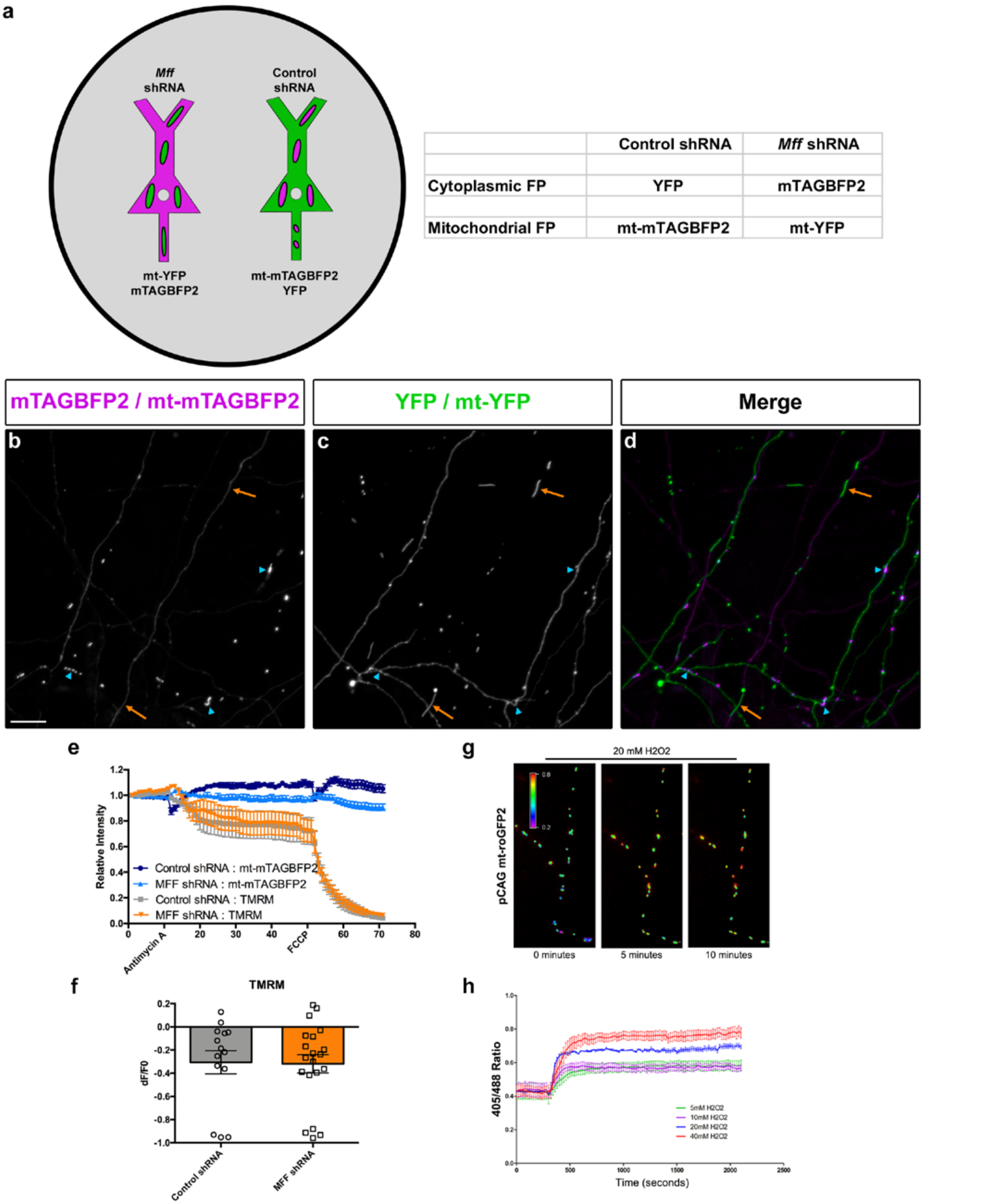

Supplementary Figure 5. Paradigm for the measure of mitochondrial membrane potential in both control and *Mff*-deficient dissociated neuronal co-culture

(**a**) Schematic representation of how neurons were labeled with color-swapped fluorescent proteins to identify axons from control and *Mff* shRNA-expressing neurons before TMRM labeling. Following EUE with DNA plasmids encoding control shRNA, mt-mTAGBFP2 and YFP or *Mff* shRNA, mt-YFP and mTAGBFP2, dissociated neurons were mixed at a 1:1 ratio and plated on coverslips for co-cultures. This strategy allows for the simultaneous imaging of mitochondrial membrane potential (TMRM-see **Fig. 5a-b**) in both control and *Mff* knockdown neurons under the exact same culture conditions. (**b**) Representative field of view for mTAGBFP2/mt-mTAGBFP2. (**c**) Representative field of view for YFP/mt-YFP. (**d**) Merged channels shown in b-c where control (sky blue arrowheads) and *Mff* knockdown (orange arrows) axons can be visualized in the same field of view. (**e**) Relative intensity of TMRM labeling or mt-mTAGBFP2 following treatment with Antimycin A and FCCP (both 1.25µM). (**f**) Change in TMRM intensity upon Antimycin A treatment. F_0 = 10 minutes at Antimycin A addition, F = 49 minutes directly before FCCP addition. Data is represented as mean ± sem. $n_{\text{control shRNA}}$ = 14 neurons; $n_{\text{MFF shRNA}}$ = 20 neurons. Mann-Whitney test; p = 0.6913. (**g**) Validation of mt-roGFP2 probe upon treatment with hydrogen peroxide (20mM). (**h**) Ratio of emission for roGFP2 following excitation at 405nm and 488nm following treatment with the specified concentration of hydrogen peroxide. Scale bars represent the following lengths: 10µm. Related to **Figure 5.**

Supplementary Figure 6

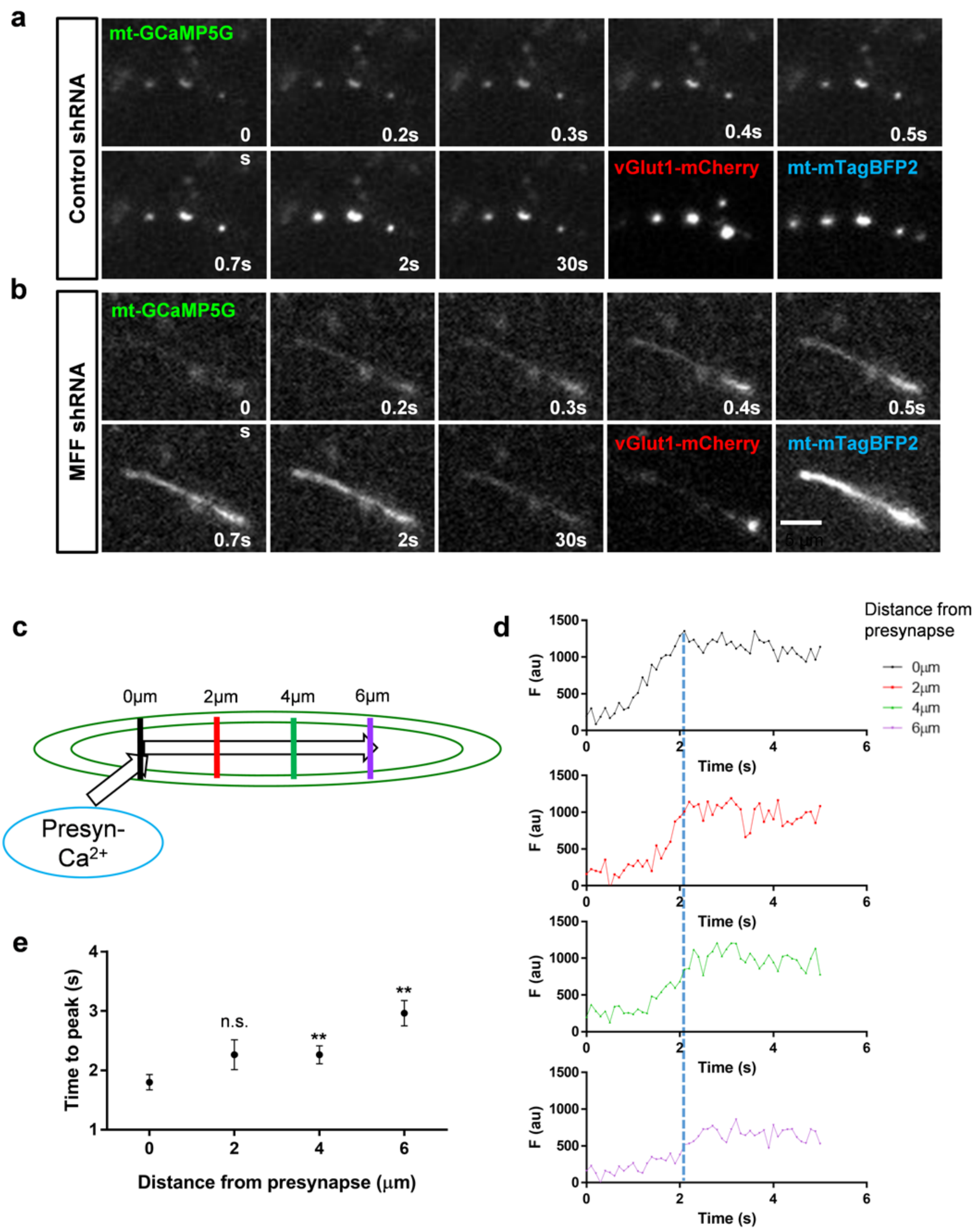

Supplementary Figure 6. Long mitochondria in *Mff* knockdown neurons show diffusion of imported Ca^{2+}

(**a-b**) Cropped mitochondrial Ca^{2+} time-lapse images of control and *Mff* knockdown axons. Presynaptic mitochondrial Ca^{2+} signals were captured with 100ms interval for monitoring diffusion of imported Ca^{2+} through mitochondrial matrix. Ca^{2+}

propagation in long mitochondria of *Mff* knockdown axons occurs from presynaptic sites. (**c**) For quantification of diffusion time, analysis was performed with single presynapse-overlapped mitochondria from *Mff* knockdown neurons. (**d-e**) Graphs display the latency of time to peak depending on distance from presynaptic sites. $n_{MFF\ shRNA}$ = 8 dishes, 8 mitochondria. p=0.0012 for 0 vs. 4, 0.0067 for 0 vs. 6. One-way ANOVA, Bonferroni's multiple comparison test. Related to **Figure 6.**

Supplementary Figure 7

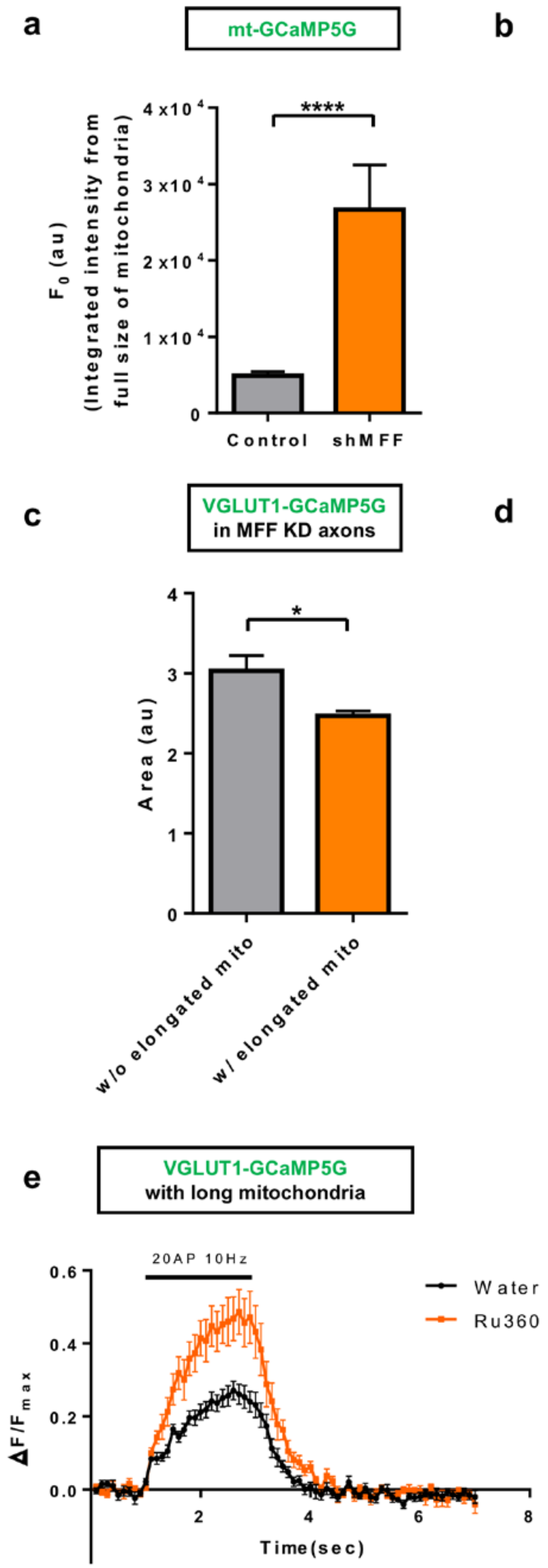

Supplementary Figure 7. Altered presynaptic functions in *Mff* knockdown neurons are mitochondria-dependent

(**a**) The basal (F_0) Ca^{2+} level integrated over the entire the mitochondrial matrix is increased in elongated mitochondria (shMFF) compared to control mitochondria (control shRNA). (**b**) However, basal average Ca^{2+} level of elongated mitochondria is lower than control mitochondrial when normalized by mitochondrial size. All graphs are represented with mean ± sem. $n_{control}$ = 7 dishes, 25 mitochondria; $n_{MFF\ shRNA}$ = 11 dishes, 14 mitochondria. (**c**) Total Ca^{2+} charge transfer (area under the curve) of presynaptic Ca^{2+} dynamics (VGLUT1-GCaMP5G at boutons associated with elongated mitochondria is lower than free boutons. $n_{w/o\ mito}$ = 31 boutons; $n_{w\ mito}$ = 37 boutons from 19 axons. p=0.0258, Mann-Whitney test. (**d**) In *Mff* knockdown axons, synaptic vesicle exocytosis (SynpH) at boutons associated with elongated mitochondria is lower than boutons not associated with mitochondria. $n_{w/o\ mito}$ = 11 boutons; $n_{w\ mito}$ = 9 boutons from 9 axons. p=0.0023, Mann-Whitney test. (**e-f**) Reduced presynaptic Ca^{2+} level (VGLUT1-GCaMP5G) at presynaptic boutons associated with long mitochondria is significantly elevated by MCU inhibitor incubation (Ru360, 10µM, 20min) compared to control (vehicle water). n_{water} = 10 boutons from 6 dishes; n_{Ru360} = 13 boutons from 6 dishes. p=0.0011, Mann-Whitney test. Related to **Figures 6 & 7.**

Supplementary Figure 8

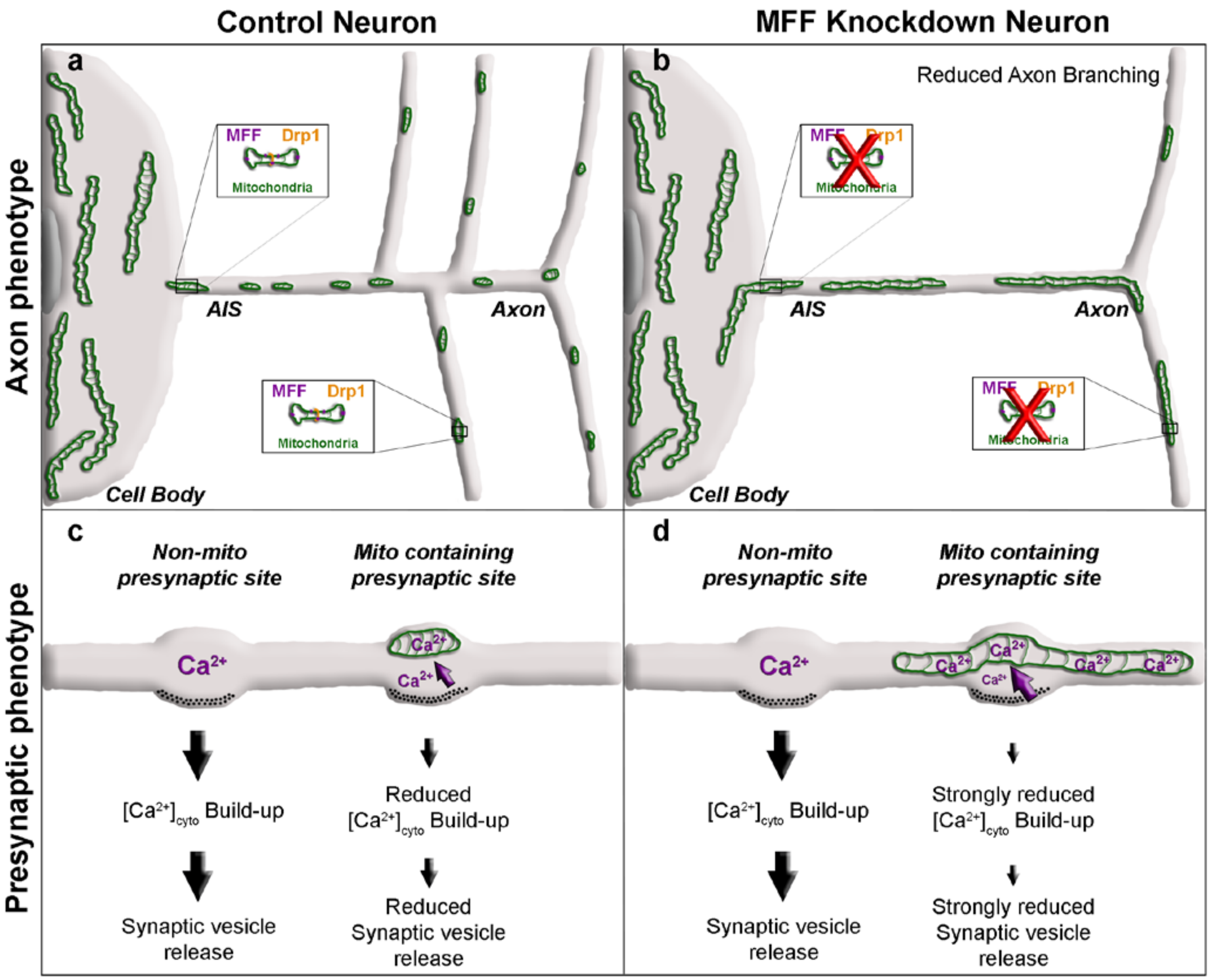

Supplementary Figure 8. MFF-dependent mitochondrial fission regulates presynaptic release and axon branching by limiting axonal mitochondria size

(**a**) In control neurons, MFF activity is required for the small size of axonal mitochondria both upon axonal entry, as well as for maintenance along the axon. Mitochondrial size is maintained by coupling the majority of mitochondrial fusion events to a fission event. (**b**) Loss of MFF activity increases mitochondrial entry size and decreases the ratio of fission to fusion along the axon. This leads to increased mitochondrial size along the axon and reduced terminal branching. (**c**) In control axons, ~50% of presynaptic boutons are occupied by mitochondria. Upon neuronal activity, the mitochondria buffer a significant amount of Ca^{2+} influx, in an MCU dependent manner, thereby reducing presynaptic vesicle release as compared to presynaptic sites without mitochondria. (**d**) Upon MFF knockdown, the increased mitochondrial matrix volume allows for increased Ca^{2+} uptake after neuronal activity, and strongly reduces presynaptic vesicle release at these sites. The reduction in terminal axon branching observed following MFF knockdown is likely due to this reduction in presynaptic release as presynaptic release is required for the stabilization of axonal branches.

Supplementary Methods

REAGENT or RESOURCE	SOURCE	IDENTIFIER
Antibodies		
Chicken anti-GFP	Aves	GFP-1020
Mouse anti-HA.11 – Clone 16B12	Covance	MMS-101R
Rabbit anti-RFP	Abcam	Ab62341
Rabbit anti-MFF	Protein Tech	17090-1-AP
Rabbit anti-ERp72 (D70D12)	Cell Signaling Tech	5033S
Kits		
Infusion HD Cloning Plus	Clontech	638911
Chemicals, Peptides, and Recombinant Proteins		
Fast Green	Sigma	F7258
Hank's Balance Salt Solution	Thermo Fisher Sci	14185-052
HEPES	Thermo Fisher Sci	15630-080
B27 Supplement	Thermo Fisher Sci	17504-004
GlutaMAX	Thermo Fisher Sci	35050-061
Neurobasal	Thermo Fisher Sci	21103-049
Penicillin/Streptomycin	Thermo Fisher Sci	15140-122
Papain	Worthington	LK003178
DNase	Sigma	D5025
Poly-D-Lysine	Sigma	P0899
Fetal Bovine Serum	Gemini Bio-Products	100-500
Normal Goat Serum	Thermo Fisher Sci	16210-064
BSA	Sigma	A7906
PBS	Sigma	P4417
NaCl	Sigma	746398
KCl	Sigma	P5405
NaH_2PO_4	Sigma	S5011
$CaCl_2$	Sigma	C5670
Glucose	Sigma	G7021
NH_4Cl	Sigma	A9434

Tetramethylrhodamine methyl ester perchlorate (TMRM)	Sigma	T5428
APV	Tocris	0106
CNQX	Tocris	1045
Ionomycin	EMD Millipore	407950
Trizma	Sigma	T1503
Trizma-HCl	Sigma	T3253
$MgCl_2$	Sigma	M4880
Protease and phosphatase cocktail inhibitors	Sigma	11836170001
Benzonase	EMD Millipore	70664-3
EDTA	Sigma	E6758
NP-40	Sigma	NP40
Triton X-100	Sigma	X100
Tween 20	Sigma	P9416
CGP 37157	Tocris	1114
Antimycin A	Agilent	103015-100
FCCP	Agilent	103015-100
Ru360	EMD Millipore	557440
Experimental Models: Cell Lines		
Human: HEK cells	ATCC	
Experimental Models: Organisms/Strains		
Mouse: CD1 IGS	Charles River Labs	Strain Code: 022
Mouse: 129-ELITE	Charles River Labs	Strain Code: 476
Mouse: C57Bl/6	Charles River Labs	Strain Code: 027
Oligonucleotides		
Mff shRNA: CCGGGATCGTGGTTACAGGAAATAACTCGAGTTATTTCCTGTAACCACGATCTTTTTG	Sigma	TRCN0000174665

Mff shRNA: CCGGCTTCATTAAGACGTCAGATAACTCGAGTTATCTGACGTCTTAATGAAGTTTTTTG	Sigma	TRCN0000174539
Control shRNA: CCGCAGGTATGCACGCGT	Moffat et al, 2006 [1]	Addgene Plasmid 10879
Recombinant DNA		
pCAG mt-YFP	Lewis et al, 2016[2]	N/A
pCAG mt-DsRED	Courchet et al, 2013[3]	N/A
pCAG mt-mTAGBFP2	Lewis et al, 2016[2]	N/A
pCAG mt-mEos2	Lewis et al, 2016[2]	N/A
pCAG far-INF	Lewis et al, 2016[2]	N/A
pCAG mt-GCaMP5G	Kwon et al, 2016[4,5]	N/A
pCAG mt-ATEAM1.03	This paper	N/A
pCAG mt-Grx1-roGFP2	This paper	N/A
pCAG vGLUT1-mCherry	Kwon et al, 2016[4]	N/A
pCAG vGLUT1-HA	This paper	N/A
pCAG vGLUT1-GCaMP5G	Kwon et al, 2016[4]	N/A
pCAG synaptophysin-mCherry	Kwon et al, 2016[4,6]	N/A
pCAG syp-pHluorin	Kwon et al, 2016[4,7]	N/A
pSCV2 mVenus	Hand et al, 2011[8]	N/A
pCAG mTAGBFP2	This paper	N/A
pCAG tdTomato	Lewis et al, 2016[2]	N/A
pLKO1.5	Moffat et al, 2006 [1]	Addgene Plasmid 10879
pCAFNF mTAGBFP2	This paper	N/A
pCAFNF mt-YFP	This paper	N/A
pCAG Flpe	Matsuda et al, 2007[9]	Addgene Plasmid 13787
pCAG Flag-hMFF	Tomaya et al, 2016[10]	N/A
pCAG HA-mMff	This paper	N/A
pCAG mScarlet	This paper	Addgene Plasmid 85042

Supplementary References

1 Moffat, J. *et al.* A lentiviral RNAi library for human and mouse genes applied to an arrayed viral high-content screen. *Cell* **124**, 1283-1298, doi:10.1016/j.cell.2006.01.040 (2006).

2 Lewis, T. L., Turi, G. F., Kwon, S. K., Losonczy, A. & Polleux, F. Progressive Decrease of Mitochondrial Motility during Maturation of Cortical Axons In Vitro and In Vivo. *Current Biology*, doi:10.1016/j.cub.2016.07.064 (2016).

3 Courchet, J. *et al.* Terminal axon branching is regulated by the LKB1-NUAK1 kinase pathway via presynaptic mitochondrial capture. *Cell* **153**, 1510-1525, doi:10.1016/j.cell.2013.05.021 (2013).

4 Kwon, S. K. *et al.* LKB1 Regulates Mitochondria-Dependent Presynaptic Calcium Clearance and Neurotransmitter Release Properties at Excitatory Synapses along Cortical Axons. *PLoS Biol* **14**, e1002516, doi:10.1371/journal.pbio.1002516 (2016).

5 Akerboom, J. *et al.* Optimization of a GCaMP calcium indicator for neural activity imaging. *J Neurosci* **32**, 13819-13840, doi:10.1523/JNEUROSCI.2601-12.2012 (2012).

6 Maximov, A., Tang, J., Yang, X., Pang, Z. P. & Sudhof, T. C. Complexin controls the force transfer from SNARE complexes to membranes in fusion. *Science* **323**, 516-521, doi:10.1126/science.1166505 (2009).

7 Miesenbock, G., De Angelis, D. A. & Rothman, J. E. Visualizing secretion and synaptic transmission with pH-sensitive green fluorescent proteins. *Nature* **394**, 192-195, doi:10.1038/28190 (1998).

8 Hand, R. & Polleux, F. Neurogenin2 regulates the initial axon guidance of cortical pyramidal neurons projecting medially to the corpus callosum. *Neural Dev* **6**, 30, doi:10.1186/1749-8104-6-30 (2011).

9 Matsuda, T. & Cepko, C. L. Controlled expression of transgenes introduced by in vivo electroporation. *Proc Natl Acad Sci U S A* **104**, 1027-1032, doi:10.1073/pnas.0610155104 (2007).

10 Toyama, E. Q. *et al.* Metabolism. AMP-activated protein kinase mediates mitochondrial fission in response to energy stress. *Science* **351**, 275-281, doi:10.1126/science.aab4138 (2016).
