# Supplementary material for: MFF-dependent mitochondrial fission regulates presynaptic release and axon branching by limiting axonal mitochondria size
Source: Nat Commun. 2018 Nov 27;9:5008. doi: 10.1038/s41467-018-07416-2 (PMC6258764; doi:10.1038/s41467-018-07416-2)
Supplement: Supplementary file 3 — Description of Additional Supplementary Files [file 41467_2018_7416_MOESM3_ESM.pdf]

Description of Additional Supplementary Files

File Name: Supplementary Movie 1
Description: **Dynamics of mitochondrial entry into the axon upon *Mff* knockdown.** In 10DIV control neurons, mitochondria enter the axon already small and at a rate of approximately eight mitochondria per hour. In *Mff* knockdown neurons of the same age mitochondria enter with a much larger size and at a reduced rate of approximately three mitochondria per hour. Related to **Figures 2 and 3.**

File Name: Supplementary Movie 2
Description: **Mitochondrial fission and fusion dynamics along the axon upon *Mff* knockdown.** In 10DIV control axons, mitochondria are maintained at a small size by regulating the fission and fusion rate to around one. Upon loss of Mff, the fission fusion rate plummets causing increased mitochondrial size along the axon. Related to **Figures 2 & 3.**

File Name: Supplementary Movie 3
Description: **Axonal mitochondrial motility upon *Mff* knockdown.** Loss of Mff does not prevent mitochondrial movement along the axon, even though mitochondrial length is strongly increased. Related to **Figures 2,3 & Supplemental Figure 4**.

File Name: Supplementary Movie 4
Description: **Presynaptic mitochondrial Ca^{2+} uptake upon evoked activity in *Mff* knockdown axons.** Elongation of axonal mitochondria upon loss of Mff increases their capacity for calcium buffering at the presynaptic site during neuronal activity. Calcium uptake is visualized via mitochondrial matrix targeted GCaMP. Related to **Figure 6.**
